# A Weekly Indicator of Surface Moisture Status from Satellite Data for Operational Monitoring of Crop Conditions

**DOI:** 10.3390/s17061338

**Published:** 2017-06-09

**Authors:** Francesco Nutini, Daniela Stroppiana, Lorenzo Busetto, Dario Bellingeri, Chiara Corbari, Marco Mancini, Enrico Zini, Pietro Alessandro Brivio, Mirco Boschetti

**Affiliations:** 1 Institute for Electromagnetic Sensing of the Environment, Italian National Research Council, Via Bassini 15, Milano 20133, Italy; stroppiana.d@irea.cnr.it (D.S.); busetto.l@irea.cnr.it (L.B.); brivio.pa@irea.cnr.it (P.A.B.); boschetti.m@irea.cnr.it (M.B.); 2 ARPA Lombardia, Lombardy Environmental Protection Agency, Via Ippolito Rosellini 17, Milano 20124, Italy; d.bellingeri@arpalombardia.it (D.B.); e.zini@arpalombardia.it (E.Z.); 3 Politecnico di Milano, Department of Civil and Environmental Engineering, Piazza Leonardo da Vinci 32, Milano 20133, Italy; chiara.corbari@polimi.it (C.C.); marco.mancini@polimi.it (M.M.)

**Keywords:** evaporative fraction, surface moisture status, corn yield, crop monitoring

## Abstract

The triangle method has been applied to derive a weekly indicator of evaporative fraction on vegetated areas in a temperate region in Northern Italy. Daily MODIS Aqua Land Surface Temperature (MYD11A1) data has been combined with air temperature maps and 8-day composite MODIS *NDVI* (MOD13Q1/MYD13Q1) data to estimate the Evaporative Fraction (*EF*) at 1 km resolution, on a daily basis. Measurements at two eddy covariance towers located within the study area have been exploited to assess the reliability of satellite based *EF* estimations as well as the robustness of input data. Weekly syntheses of the daily *EF* indicator (*EF_w_*) were then derived at regional scale for the years 2010, 2011 and 2012 as a proxy of overall surface moisture condition. *EF_w_* showed a temporal behavior consistent with growing cycles and agro-practices of the main crops cultivated in the study area (rice, forages and corn). Comparison with official regional corn yield data showed that variations in *EF_w_* cumulated over summer are related with crop production shortages induced by water scarcity. These results suggest that weekly-averaged *EF* estimated from MODIS data is sensible to water stress conditions and can be used as an indicator of crops’ moisture conditions at agronomical district level. Advantages and disadvantages of the proposed approach to provide information useful to issue operational near real time bulletins on crop conditions at regional scale are discussed.

## 1. Introduction

Early warning systems able to exploit indicators of cropland status are essential sources of information to improve yield forecasts and resource management [1]. Sustainable management at regional scale of water resources relies on crop water needs information, especially over scenarios of climate change in anthropized regions where water is over exploited and shared among multiple usages.

Since more than 80% of the water applied to agricultural lands can be consumed by evapotranspiration (ET) [2], monitoring temporal and spatial ET variations is necessary. Traditional methods for direct or indirect in situ measurement of ET, such as lysimeters, sap flow meters and eddy covariance towers, provide accurate point and/or parcel-scale values but spatially distributed information at province/regional scale can hardly be extrapolated due to heterogeneity of land surface and complexity of the hydrological processes [3].

Since Remote Sensing (RS) data can provide a large variety of information on crop status and surface hydrological conditions, which are of key importance to highlight potential criticalities to support water planning and provide quantitative data for management [4,5], RS techniques are recognized as the only viable means to map ET at regional scale in a consistent and economically feasible way [6]. Instantaneous values of ET at satellite overpass can be used as diagnostics for surface status [7], or as controls for hydrological models through assimilation schemes [8]. Several approaches have been developed to estimate ET and/or indicators of water stress, spanning from simple empirical methods to more complex energy balance models, as fully described in literature reviews [9,10,11,12].

### 1.1. The Triangle Method: A Short Review on Past Applications and Recent Improvements

Among the available RS-based approaches for ET analysis, the triangle method, based on the work of Price in the early 1990s [13] and later elaborated by Jiang and Islam [14,15,16], has been widely exploited to estimate the Evaporative Fraction (*EF*), which is the ratio between the latent heat flux and the total available energy at canopy surface [5,17]. *EF* is both a key parameter to estimate ET as well as a direct indicator of surface moisture conditions [18] and water stress itself [19,20]. This approach, regarded as a simplification of more complex models such as the Surface Energy Balance Algorithm for Land (SEBAL) [21], is suitable for large area monitoring of surface moisture conditions [14,22]. Among its advantages are the simple parameterization/calibration and its reliance on operational satellite data [6,20,23], which allow to spatially explicitly estimate EF in near real-time over large areas.

In its original formulation, the triangle method builds on the triangular shape of the scatter plot of remotely sensed surface/canopy temperature (*T_s_*) versus a Vegetation Index (VI) such as the Normalized Difference Vegetation Index (*NDVI*) [10,24]. This scatterplot is used to compute the so-called wet and dry edges: the wet edge corresponds to areas where *EF* is at maximum (i.e., maximum evapotranspiration) whereas the dry edge corresponds to areas where the *EF* is close to zero (i.e., limited water availability) [23]. Given two co-registered raster images containing temperature and VI value, *EF* can then be estimated for each pixel based on its relative position with respect to the two edges (see Section 3.1 for details).

Alternative formulations of the method were also proposed in later studies. In particular, using the difference between air surface and air temperature (*ΔT* = *T_s_* − *T_a_*) instead than simply *T_s_* to build the aforementioned scatterplot was proposed by Moran et al. [25] and Jiang and Islam [16], and successively adopted by numerous studies (e.g., [26,27,28,29]). Additionally, other RS-derived indicators of vegetation characteristics (e.g., fractional cover, albedo, etc.) were proposed as an alternative to NDVI for computing the dry and wet edges [6,30].

The triangle method has been exploited starting from data acquired by several satellite platforms/sensors, among which NOAA-Advanced Very High Resolution Radiometer (AVHRR) [31], Meteosat Second Generation—Spinning Enhanced Visible and Infrared Imager (MSG SEVIRI) [32], NASA Moderate Resolution Imaging Spectroradiometer (MODIS) [2], Envisat-Advanced Along-Track Scanning Radiometer (AATSR) and Medium Resolution Imaging Spectrometer (MERIS) [23], Landsat Thematic Mapper/Enhanced Thematic Mapper (TM/ETM+) [23] as well as airborne sensors [28]. One of its main advantages is its suitability for hydrological studies where field data availability is limited or missing. Thanks to this, it was implemented in operational monitoring systems such as the EVapotranspiration Assessment from SPAce (EVASPA) tool [33], exploited to derive the Temperature Vegetation Dryness Index (TVDI) [25] used for vegetation water stress detection [34,35,36,37] and soil moisture estimation [38,39] in semi-arid, temperate and tropical areas [18] and used to improve the performances of hydrological models [40].

Nevertheless, it is important to remind that several issues should be addressed for a full exploitation of satellite based EF/ET estimations.

A first assumption of the method is that incoming energy, aerodynamic properties and atmospheric conditions have to be reasonably uniform over the area at the time of satellite overpass [2,23,32] and the AOI should be as flat as possible in order to identify a proper triangular shape in the pixel distribution [24]. Tave et al. [41] recently tried to solve this requirement by improving the triangle method to compute EF for different elevation zones rather than keeping the estimation of flat area only.

Second, a key issue for applying the triangle method is the proper computation of the wet and dry edges, which influences the accuracy of EF estimation [6]. The method can therefore be applied only under specific conditions, in relation to study area characteristics and EO data exploited, and a proper data handling is needed to retrieve reliable estimates. In particular: (i) a large range of combined soil moisture status and vegetation characteristics must be present in the study area to represent a wide range of evapotranspiration conditions [37,42], and (ii) this variability must be properly captured by the RS data used. These conditions influence the required extension of the area of interest (AOI) analyzed, which should be sufficiently large [43] to allow the construction of reliable scatterplots as a function of the spatial resolution of the EO data used. According to previous studies, the triangular shape depends more on the number of pixels, rather than on the spatial resolution [24,44]. Hence, for small AOIs (e.g., ~4000 km^2^) HR satellite data may allow obtaining more accurate EF estimations. On the other hand, while working with satellite data at coarse resolution the AOI should be bigger since a larger area is needed for an appropriate definition of the triangle shape [23].

If these conditions are not completely fulfilled, the shape of the temperature vs. VI scatterplot may be “flawed” (i.e., not triangular at all), leading the estimated dry and wet edges to be far from the theoretical ones, and therefore to large inaccuracies in EF estimation [32].

To deal with the aforementioned problem, many authors proposed modifications and advancements to the “standard” method allowing a better and more stable computation of the edges. Tang et al. [6] proposed a specific pre-processing technique for identifying outliers in the temperature vs. VI scatterplot space and removing them before the computation of the edges. De Tomas et al. [23] demonstrated how this approach can improve EF estimates when HR data (e.g., Landsat like) are used. Maltese et al. [28] compared scatterplots derived from different dates suggesting that, to cope with the uncertainness in the dry and wet edges identification, a multi-temporal analysis should be exploited to include outer extremes in soil water content. Minacapilli et al. [2] further developed this idea building the temperature vs. VI scatterplot in the temporal domains for each pixel of the AOI using all available dates; application of this approach resulted in an accurate estimation of ET at regional scale. Other recent contributions determined the theoretical edges on the base of the surface energy balance principle rather than by identifying the boundaries empirically in the data space; EF is then estimated for each pixel using the observed temperature vs. VI scatterplot [22,32]. Comparison with in situ data of soil moisture and EF revealed that this approach could increase the accuracy of the triangle method. However, its implementation requires a more complex parameterization and requires input field data not be always available over the AOI.

Another important issue discussed in literature is whether the output of the triangle method can be considered representative of the daily condition or the instantaneous momentum. Most approaches based on satellite data assumed EF to be relatively constant in daytime (“self-preservation of EF” [23]), so that satellite-based instantaneous EF estimates can be representative of daily conditions [30] (see Section 3.1 for details).

Nevertheless, for some scientific applications it is necessary to more rigorously upscale the instantaneous estimation to daily conditions taking into account the diurnal cycle using exogenous information such as meteo data [45]. To perform this daily upscaling, Trezza [46] proposed to multiply the instantaneous EF by the daily reference ET computed with FAO approach [47] while Ryu et al. [48] exploited the variations of the daily extraterrestrial solar radiation. Finally, Tang et al. [49] recently presented a promising approach for upscaling, based on half-hourly meteo data (i.e., air temperature, wind speed, vapor pressure, and surface available energy). According to the authors, this allows reducing the underestimation of EF typically obtained using the “constant EF” assumption [50].

Finally, it is important to remind that, being based on optical RS data, EF time series often present gaps due to cloud cover. Therefore, if complete and continuous (e.g., daily) EF estimates are needed (e.g., for assimilation in hydrological models) appropriate gap filling approaches should be implemented. For example, Rasmussen et al. [27], developed a specific solution to deal with this problem, allowing to obtain a complete daily coverage of EF at regional scale.

### 1.2. Study Contribution and Objective

The previous review highlighted the maturity of surface moisture status assessment from satellite data, and that the triangle method can be applied operationally for a study area with specific characteristics, if performing a proper data analysis. In this framework, this paper aims to develop an operational approach, based on the triangular method, to derive a weekly spatially explicit indicator of crop surface moisture conditions at regional scale. The implemented solution produces weekly EF map over the agricultural areas of Lombardy, Northern Italy, covering more than 1,200,000 ha, starting from moderate resolution daily EO data from MODIS sensor. *EF* estimates were produced for the years 2010 to 2012 and locally validated with in-situ eddy-covariance data. The utility and robustness of the proposed indicator for operational monitoring of crop conditions at regional scale was assessed by comparing seasonal synthesis of *EF* and *NDVI* with official yield data.

## 2. Materials

### 2.1. Study Area Description

**Figure 1:**
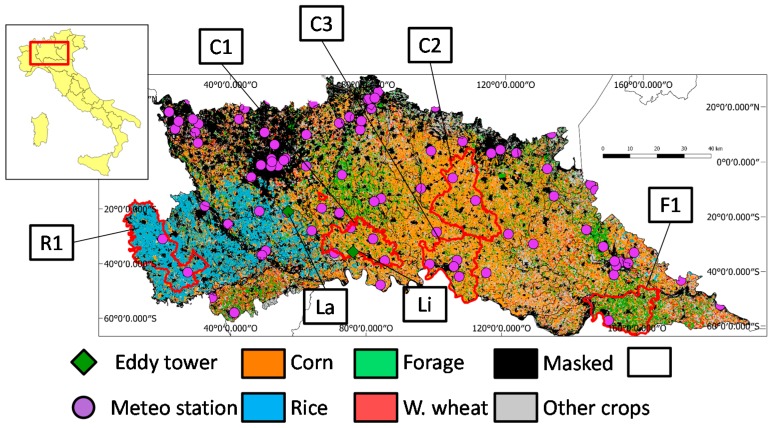
Distribution of the four major crop types in the 2011 summer season in the study area with the location of the meteorological stations (purple dots) and eddy towers at Landriano (La) and Livraga (Li) (green diamonds). Black polygon boundaries divide the region by its main agronomical districts, with the red ones highlighting six districts of interest for this paper (3.2.2) corresponding to: a rice district (R1: Western Lomellina), 3 Corn districts (C1: Codogno, C2: Western Bresciana and C3: Cremona) and a forage district (F1: Western Oltrepo Mantovano).

The study was focused on agricultural areas of Lombardy, in Northern Italy. The AOI is a part of Po valley and spans about 12,000 km^2^. Topography of the study area is mostly flat, with altitude slowly degrading from about 150 m in the North West to about 20 m in the Eastern area. It is intensively cultivated with summer crops (i.e., corn (*Zea mays* L.), forage and rice (*Oryza sativa* L.)) and winter wheat (*Triticum* L.), which cover about 68% and 8% of the agricultural land, respectively. It is divided in 45 agronomical districts, showing marked differences in the main cultivated crops (Figure 1).

Average air temperature is 13–14 °C, with summer (June-August) and winter (December-February) average values of 23–24 °C and 2–3 °C, respectively [51]. Precipitations are concentrated in autumn and spring, with a mean annual total of 800 mm. In this region, which is one of the most urbanized and industrialized of Italy, water management is critical during the dry summer months when multiple and conflicting usages (i.e., civil, industrial, agriculture and hydroelectric) can reduce water availability for irrigation. Official statistics of annual crop yield over the agricultural districts are publicly made available by the Lombardy Environmental Protection Agency (ARPA).

As the area shows flooded, irrigated and not-irrigated crops, is considered suitable for regional monitoring of crop/surface moisture conditions with the triangle approach, which requires a large number of pixels to cover a wide range of soil moisture and vegetation conditions [11,23]. In the last decade, the area experienced water scarcity in summer months when water availability could not satisfy the multiple water requirements [52] due to a lack of precipitations in key periods of crops growing cycle and/or reduced snowfall in winter. Irregular precipitations might also be accompanied by excessively high temperatures, such as in the year 2003, when corn production in Italy was 36% lower than the 1998–2002 average [53]. Besides climatic conditions, non-optimal timing in water supply to farmers by public authorities can worsen crop conditions, as in 2012 when corn production decreased by about 15–20% due to water shortage [54]. The reduction in corn production was worsened by the outbreak of aflatoxin contamination in corn stocks [55] induced by the occurrence of water stress conditions during critical phenological stages.

To face these problems, regional authorities involved in agro-monitoring over this area require therefore detailed information about the temporal and spatial variations of crop conditions. Although periodical agro-meteorological bulletins which analyze the impact of water stress on crop production are already issued for the study area by international agencies (e.g., Joint Research Centre-Monitoring Agricultural Resources Unit—JRC-MARS [56]; Global Agricultural Monitoring Initiative—EOGLAM [57]), they are not sufficiently detailed in terms of spatial resolution. More specific regional monitoring programs providing dedicated analyses for the agricultural areas of Lombardy are therefore needed.

### 2.2. Satellite Data and Preprocessing

MODIS 250 m resolution 16-day Vegetation Indices (VI) composites (Products MOD13Q1/MYD13Q1) and 1 km Land Surface Temperature and Emissivity (LST/E) daily (Product MYD11A1) data and the corresponding Quality Assurance (QA) information [58] over tile h18v04 and the period 2010–2012 have been downloaded and pre-processed using the MODIStsp “R” package [59].

Concerning vegetation indexes data, the use of composite data allowed to exploit VI already derived from the best possible observations in the composite period, thus reducing oscillations related to varying observation geometries and atmospheric conditions. Moreover the synergic use of Terra and Aqua products VIs, with a theoretical 8 days shift, allowed to analyze a denser time series.

The raw M*D13Q1 time series were smoothed in order to remove residual noise in the *NDVI* signal, using a two-step approach based on (i) outlier removal and (ii) weighted Savitzky-Golay filtering with weights assigned on the basis of QA information, considering the real date of observation derived from the Day of Acquisition MODIS layer. In particular, weights are computed based on pixel reliability and VI usefulness indicators derived from the MODIS QA layers, complemented with specific thresholds blue reflectance [60,61]. The algorithm output consists in a smoothed 250 m *NDVI* time series with an 8-day regular time step.

This product was finally resampled to 1 km spatial resolution through average aggregation, and daily time series were generated through linear interpolation between values of the nearest dates. This allows to reduce potential biases during the periods of rapid crop growth.

The Aqua MYD11A1 LST/E product provided 1 km daily surface temperature (*T_s_*) accompanied by pixel-level QA flags. QA parameters were summarized in a three-level categorical Quality Flag (QF): QF1 (best quality) if all QA parameters reported the highest quality; QF0.5 (intermediate quality) if at least one of the quality layers reported a sub optimal estimation; and QF0 (bad quality) for not computed *T_s_*. QF layer was first exploited to investigate the influence of QA level on the accuracy of MODIS *T_s_* and afterwards to discard low quality pixels [62]. No smoothing process was applied to *T_s_* time series since small daily fluctuations of surface temperature may be related to actual changes rather than noise and a noise-reduction technique might cause loss of valid data. Moreover, temporal composites of *T_s_* cannot be used because the triangle method relies on the assumption of uniform atmospheric conditions and incoming energy over the study area, which cannot be assured over longer time periods [23].

### 2.3. Meteorological Data

#### 2.3.1. Air Temperature Data from Meteorological Stations

Seventy-five automatic meteorological stations belonging to the Lombardy region network (*Rete Regionale di Rilevamento Meteorologico*) and managed by the Lombardy Environmental Protection Agency (ARPA Lombardia) are located within the study area (purple dots in Figure 1). Measurements acquired by the meteorological network are publicly available through the ARPA web site (http://goo.gl/zqkmf0—last access May 2017). In particular, air temperature (*T_a_*) is available with half-hour frequency. Daily *T_a_* measurements simultaneous with Aqua satellite overpass (1.30 pm) for the 2010–2012 period were downloaded and spatially interpolated the study area by applying an Inverse Distance Weighted (IDW) approach to derive daily 1 km maps.

#### 2.3.2. Eddy Covariance Measurements

Two Eddy Covariance (EC) towers are located within the study area, respectively in Landriano (45.32 N; 9.27 E, 89 m a.s.l.) and Livraga (45.19 N, 9.57 E, 61 m a.s.l.) (Figure 1—green diamonds). The towers are located within corn fields of about 10 and 12 ha [63,64] (see Appendix A). They are equipped with a three-dimensional sonic anemometer to measure sonic temperature and the three components of wind speed, an open-path gas analyzer to measure water vapor and carbon dioxide concentrations, a net radiometer and a thermo-hygrometer. Soil moisture, soil temperature and soil heat flux observations are also measured at different depths (10–30–50 cm, 4–8 cm and 6 cm respectively) every 30 min.

Data collected for the years 2010–2011 at Landriano and 2010–2012 at Livraga were used as ground truth information. In particular, air and surface temperature measurements at the EC towers were used to assess the accuracy of the *T_a_* and *T_s_* datasets used as inputs to compute *EF*, while *EF* estimates simultaneous to the Aqua satellite overpass (1.30 pm) were compared to satellite-based *EF* estimates.

To compute *EF* at the towers location, raw EC data were corrected to account for instrumental (e.g., axis rotation for tilt corrections, spike removal, time lag compensation) and physical errors (spectral information losses, air density fluctuations and air humidity effects on sonic temperature) following procedures from the literature [65], as implemented in the PEC (Polimi Eddy Covariance) software [64]. After these corrections procedures, the energy budget has been analyzed, showing a closure ranging between 0.92 and 0.97. According to literature, a lack of energy balance closure is common in EC measurements [66,67], and can be considered acceptable up to values of 0.6 [68]. Following the procedure developed by Twine et al. [69], latent and sensible heat fluxes were analyzed respecting the Bowen-ratio method to reach the energy balance closure. Net radiation (R_n_), latent (λE), sensible (H) and soil (G) heat fluxes were then derived from corrected EC measurements and exploited to compute *EF* with a semi-hourly time step using the formulation of the energy balance [70]:

(1) $$EF=\frac{\lambda E}{\lambda E+H}=\frac{\lambda E}{R_{n}-G}$$

### 2.4. Ancillary Datasets

The *Carta Uso Agricolo Annuale* (CUUA) is a 20 m resolution land-use map produced each year by the Regional Agency for Agriculture and Forest Services of Lombardy (ERSAF) (http://goo.gl/GJPU3U, last access May 2017) by combining farmers annual declarations with the official regional land use map (*Destinazione d’Uso dei Suoli Agricoli e Forestali*—DUSAF). The map includes 21 agricultural land-cover/use classes of which the most common over the study area are shown in Figure 1. The CUUA map was used to create a 1 km mask of arable land, by retaining only pixels including at least 60% of crop land and an average elevation below 100 m a.s.l. (corresponding to maximum elevation of irrigation systems in Lombardy plains). The arable land mask was used to discard from the analysis non-agriculture (e.g., urban, forest) and mixed land-use pixels (corresponding to about 37% of the area), which inclusion would have affected *EF* retrieval performance [23].

## 3. Methods

### 3.1. Computation of EF from MODIS Data

**Figure 2:**
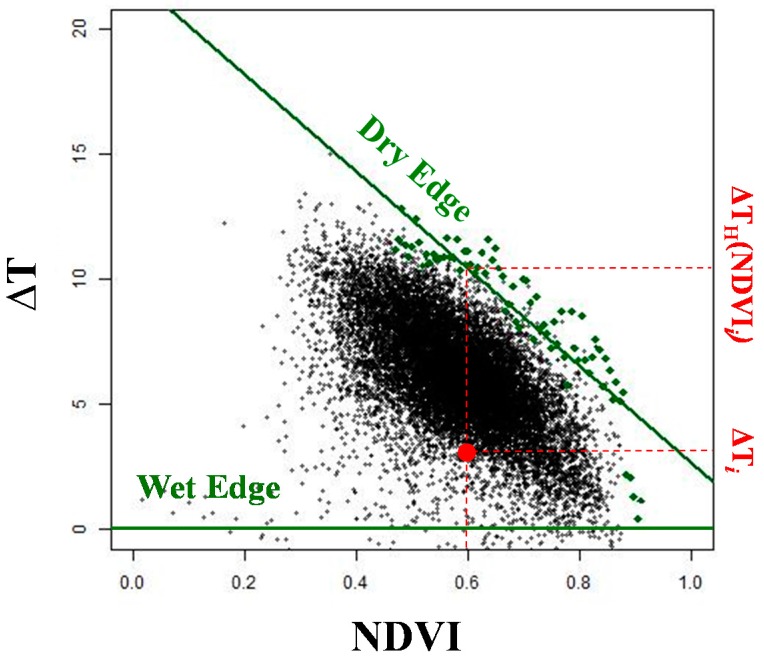
Example scatter plot between *NDVI* and *ΔT* (*T_s_* − *T_a_*) for a given day. Green dots are maximum *ΔT* values for each *NDVI* interval, green lines are the dry (wet) edges, *∆T_H_*(*NDVI_i_*) and *ΔT_i_* represent the values used in the calculation of the *EF* for the pixel *i*.

Methods for computing *EF* from satellite data generally rely on the evidence that the combination of *T_s_* and *NDVI* is diagnostic of surface moisture condition [37]. In particular, in this study we used the approach proposed by Jiang and Islam [16], which is based on the relationship between *NDVI* and the air-surface temperature difference (*ΔT* = *T_s_* − *T_a_*). The *ΔT* vs. *NDVI* bidimensional space exhibits for a given day a triangular shape, bounded by the wet (maximum evapotranspiration) and dry (minimum evapotranspiration) edges [71] (Figure 2). The wet edge position corresponds to the x-axis (*ΔT* = 0) as established by the formulation of the latent heat flux [16]. The dry edge position is instead estimated as the regression line of maximum observed *ΔT* at discrete intervals of *NDVI* (green dots in Figure 2).

Given the position of a generic point *i* in the scatterplot, *EF* is estimated on the basis of its distance from the dry and wet edges [14]:

(2) $$EF_{i}=\left(\Delta T_{H}\left(NDVI_{i}\right)-\Delta T_{i}\right)/\left(\Delta T_{H}\left(NDVI_{i}\right)\right)$$

where *ΔT_i_* is the difference between surface (*T_s_*) and air (*T_a_*) temperature for pixel *i*; $\Delta T_{H}\left(NDVI_{i}\right)$ is the *ΔT* of the dry edge in correspondence of the *NDVI* value of pixel *i*. Therefore, the closer the pixel is to the dry edge, the lower is *EF* (down to zero), meaning that it is characterized by low moisture conditions.

The described method was used to create daily 1 km *EF* maps ($EF_{d}$) over the study area for 2010–2012. Daily *NDVI* values, derived as described in (Section 2.2), and daily *ΔT* maps derived as the difference between MODIS *T_s_* maps (Section 2.2), and *T_a_* maps derived from weather stations (Section 2.3.1) were used to build the *ΔT*. vs. *NDVI* daily scatterplots, and successively derive daily *EF* values for each (cloud free) pixel. Computation was not conducted on dates with more than 40% of cloud cover since they wouldn’t include the full range of variability of thermal and crop cover conditions that is one of the essential element to reliably compute the dry and wet edges and therefore to apply the triangle method. To avoid problems due to residual cloud contamination, pixels below the lower 15th percentile of the *NDVI* distribution were not considered in the dry edge computation. The number and positions of *NDVI* intervals used to compute the regression line were determined with a logarithmic function, as proposed by Sturges [72].

Equation (2) provides instantaneous *EF* estimation at the time of satellite overpass. Although the diurnal behavior of *EF* exhibits a typical light concave-up shape with a minimum around noon [73], its variations are much lower compared to latent and sensible heat fluxes under clear sky conditions [17,73,74]. *EF* is therefore frequently assumed to be reasonably constant during daytime, and satellite-based EF estimates are often considered representative of overall daily conditions [23]. Peng et al. [17] added that the assumption of daytime constant *EF* is more robust if the estimation is performed around midday (as the case of MODIS Aqua products) and in clear sky conditions only (as is the case after the removal of contaminated data thanks to QF). Therefore, given that the aim is to estimate a weekly indicator of surface moisture conditions rather than to quantitatively estimates daily water needs, satellite based instantaneous *EF* maps were considered a suitable indicator of surface moisture conditions over large areas.

*EF_d_* maps were averaged to derive a weekly indicator of crop water stress (*EF_w_*). The use of weekly averages is more suitable for regional crop monitoring since it reduces spatiotemporal gaps due to cloud cover and it decreases the influence of day to day variability due to external factors, e.g., wind speed and other atmospheric interferences [75]. A weekly indicator is also better suited for providing information for agro-meteorological bulletins, which are usually issued with a monthly or biweekly frequency during the crop season.

### 3.2. Accuracy and Usefulness Analysis

#### 3.2.1. Comparison with In Situ Data

Comparison with in-situ data collected at the EC towers was conducted to verify the accuracy of input temperature maps (*T_a_* and *T_s_*), and *EF_w_* estimates over the 1 km pixels corresponding to the two EC towers. In the case of *EF*, the weekly average was computed starting from the daily half-hourly EC estimations obtained at the hour of satellite overpass (13:30).

Averaging over the week allows the reduction of the impact of missing measurements due to instrumental and physical errors in data acquisition. Linear regression parameters (slope, intercept and coefficient of determination r^2^) and indices of agreement (Mean Error (ME), Root Mean Squared Error (RMSE) and Relative Root Mean Squared Error (rRMSE)) were considered as performance indicators.

The comparison was limited to the growing period of the more common summer crops, i.e., between sowing (mid-April) and senescence (mid-August). Outside this period the estimation of *EF* is not reliable because the full range of thermal and vegetation cover conditions needed to calculate the dry edge are not fulfilled [6,23,32,76]. Moreover, frequent cloud cover in winter and autumn may prevent the use of satellite data for operational monitoring. Moreover, in the study area, information on crop conditions are necessary for water management and irrigation planning only in the hot and dry summer months, when water availability is a key issue. On the contrary, the autumn/winter season is not crucial for operational crop monitoring.

#### 3.2.2. Comparison to Crop Yield Statistics

The usefulness of *EF_w_* as a spatially distributed indicator of surface moisture conditions was assessed over five agronomical districts of the study area. The selected districts are representative of the major crop types: corn (C1: Codogno plain, C2: Central Bresciana plain, and C3: Cremona plain), rice (R1: Western Lomellina) and forage (F1: Western Oltrepo Mantovano). *EF_w_* profiles over these five districts were analyzed to investigate whether the indicator (i) depicts the expected trend of crop seasonal dynamics in relation to known crop characteristics and agro-practices, and (ii) provides evidence of inter-annual variability related to water stress.

Corn districts C2 and C3 witnessed a significant decrease in production in 2012, whereas corn district C1 did not suffer from water stress due to sufficient irrigation. Cumulated summer *EF_w_* (June to August) over these three districts was calculated to better investigate whether *EF_w_* estimates can provide insights on the occurrence of summer droughts and their impact on crop production. Cumulated *NDVI* over the same period was also computed since it is a proxy of vegetation biomass frequently used to identify crop production shortages [77]. One-way analysis of variance (ANOVA) followed by the post-hoc Tukey HSD (Honestly Significant Difference) [78] multiple range test were carried out over the three districts in order to evaluate the diagnostic capability of both indicators in detecting differences between the years. Inter-annual variability of the indicators was finally compared with official statistics of corn yield at district level.

## 4. Results and Discussion

### 4.1. Comparison of Air and Surface Temperature with In Situ Measurements

**Figure 3:**
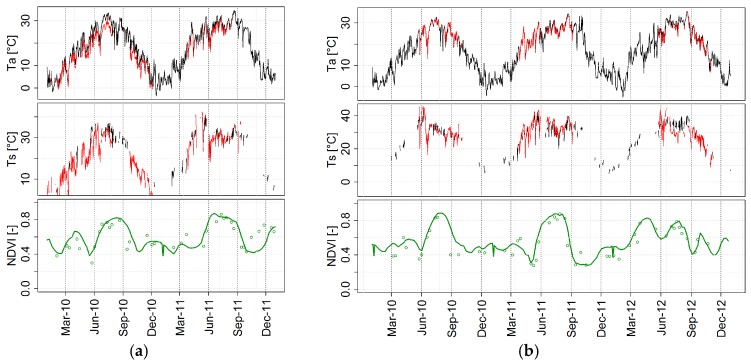
Time series of *T_a_*, *T_s_* and 250 m MODIS NDVI (dots for raw 8-day composite and daily smoothed) for the Landriano (**a**) and Livraga (**b**) sites. Black (red) lines show satellite/meteo (in situ) values.

Figure 3 shows a comparison between time-series of *T_s_* and *T_a_* estimated as described in Section 2.2 and Section 2.3.1 at the EC towers of Landriano and Livraga, and the corresponding measured values. Maximum *T_s_* and *T_a_* (about 30 and 35 °C, respectively) occur in the middle of July. This corresponds to the end of the vegetative period [79], as highlighted by the peak of the 250 m resolution *NDVI* time series (Figure 3—bottom panel).

**Figure 4:**
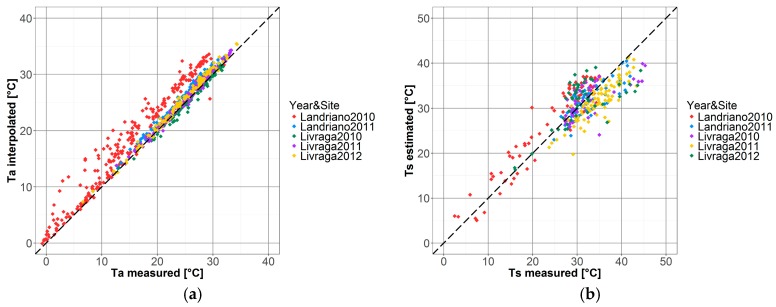
Scatter plots of daily *T_a_* (**a**) and *T_s_* (**b**) from EC measurements (x-axis) and estimated or satellite-based (y-axis) for all towers and years as highlighted by the different colors/markers. In (**b**) only data with good quality flag (QF1) are plotted.

The reliability of the exploited *NDVI*, showed by the smoothed lines in Figure 3, is significantly increased by applying the multi-temporal smoothing process described in Section 2.2; yet this kind of filter cannot be used to *T_a_* and *T_s_* time series since daily fluctuations may be related to changes in surface/air temperature and a noise-reduction technique might cause a loss of valid information. Scatter plots between in situ and estimated air and surface daily temperature are shown in Figure 4 and regression parameters are summarized in Table 1. Globally, estimated *T_a_* shows a very good accordance with data measured at the EC towers (r^2^ = 0.95; ME = 1.37) with the exception of the Landriano site in 2010 (red dots in Figure 4a), which shows a noticeable overestimation (ME = 2.94, Table A1 in Appendix B). This good agreement is favored by the spatial homogeneity of air temperature over flat areas such as the Lombardy plain [80]. Moreover, both towers are surrounded by a very dense meteorological network with at least five stations in a range of 15 km around each tower (purple dots in Figure 1) thus making *T_a_* interpolation robust.

The comparison of satellite *T_s_* vs. EC measurements showed less satisfactory results, highlighting a consistent underestimation. The analysis of the impact of the quality of satellite observations, as reported in the QA layer of the satellite datasets (Section 2.2), on the accuracy of estimated surface temperatures highlighted that data flagged as good quality (QF1) provide a significantly better estimation (ME = −0.48 °C, r^2^ = 0.74) compared to QF0.5 data (ME = −2.38 °C, r^2^ = 0.74). This testifies that QF0.5 data, although being considered as intermediate quality, are still strongly affected by residual cloud cover and/or atmospheric interference and led us to decide to retain only QF1 data for *EF_d_* computation (See Table A1 in Appendix B for a more detailed analysis on *T_s_* data quality through sites and years).

This decision caused a reduction of the cardinality of available *T_s_* estimates: for example, on the two pixels corresponding to the towers, out of the 1825 available dates only 36% was assigned to the higher quality level (QF1). This percentage raises to about 53% on summer months, suggesting that a much higher reliability can be achieved in *EF_d_* estimation in that period (See Figure A3 and Figure A4 in Appendix B for a more detailed analysis on temporal variability of *T_s_* data quality).

### 4.2. Comparison of EF_w_ with In Situ Data

**Figure 5:**
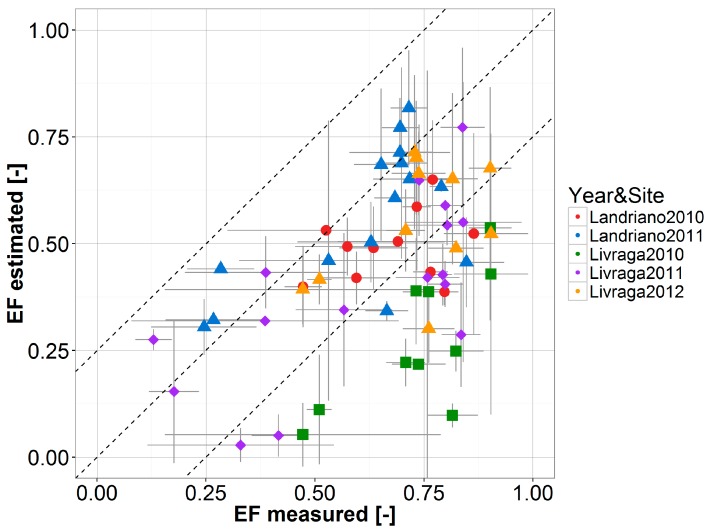
Scatterplots of average weekly *EF* derived from EC measurements (x-axis) and satellite estimation (y-axis) for all EC towers and years as highlighted by the different colors/markers. Dashed lines indicate the 1:1 line and discrepancies between estimated and observed *EF_w_* less than 0.2. Grey lines depict the average plus/minus one standard deviation ranges of the valid daily EF satellite/EC estimates available for the week.

Scatterplots comparing *EF_w_* estimates derived from satellite and from EC data are shown in Figure 5, grouped by site and year; agreement metrics are summarized in Table 2.

Satellite *EF_w_* values are generally lower than EC derived *EF_w_*, with an overall ME of −0.15, best (worst) performance are observed over Landriano 2011 (Livraga 2010). These results are consistent with Lu et al. [81], but opposite in sign to those obtained by de Tomás et al. and Peng and Loew [23,82]. This uncertainty in *EF* estimation may be related to the difference between the 1 km pixel size of MODIS maps used as inputs and the footprint of the EC towers (120 m^2^ in Landriano and 140 m^2^ in Livraga) [64]. The area surrounding the towers is fragmented and heterogeneous in terms of crop type: on average, corn covers 36% and 21% of the 1 km MODIS pixels at Landriano and Livraga, respectively. In these conditions, the 1-km *T_s_* and *NDVI* values derived from MODIS are influenced also by the characteristics of the areas surrounding the EC towers, thus making the comparison with field-estimated EF difficult.

**Figure A1:**
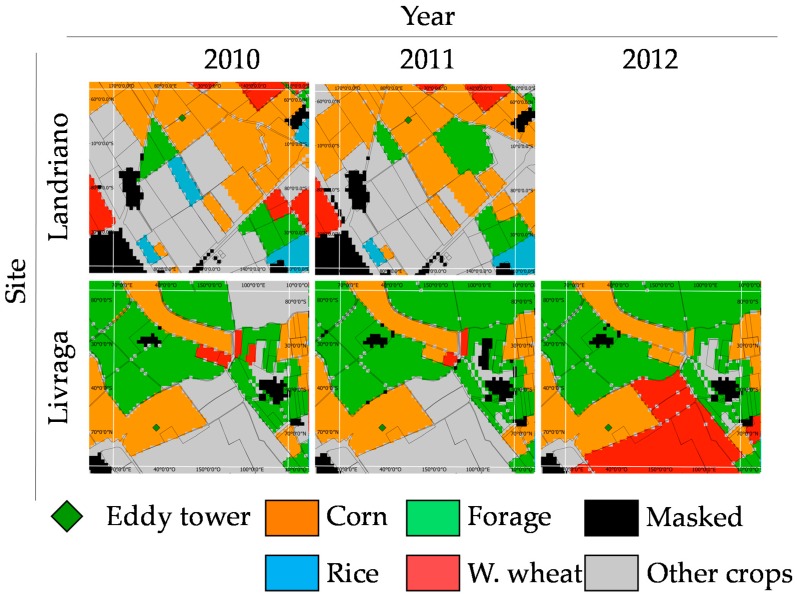
Zoom of the CUUA maps on the 1 km pixels (white squares) in correspondence of the eddy towers for the analyzed years.

**Figure A2:**
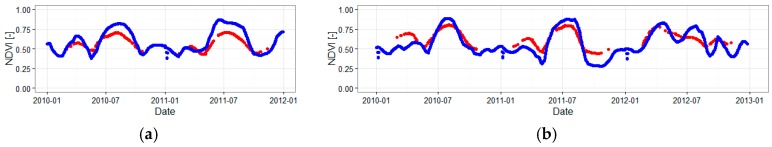
Comparison between 1 km *NDVI* (red line) and 250 m (blue line) in Landriano (**a**) and Livraga (**b**).

**Figure A3:**
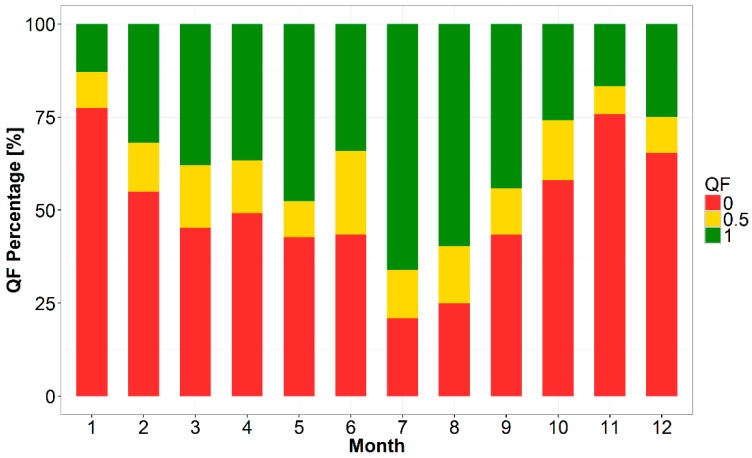
Presence in percentage of the three QF (QF1 best quality, QF0.5 intermediate quality, QF0 bad quality) over the two eddy towers for months.

**Figure A4:**
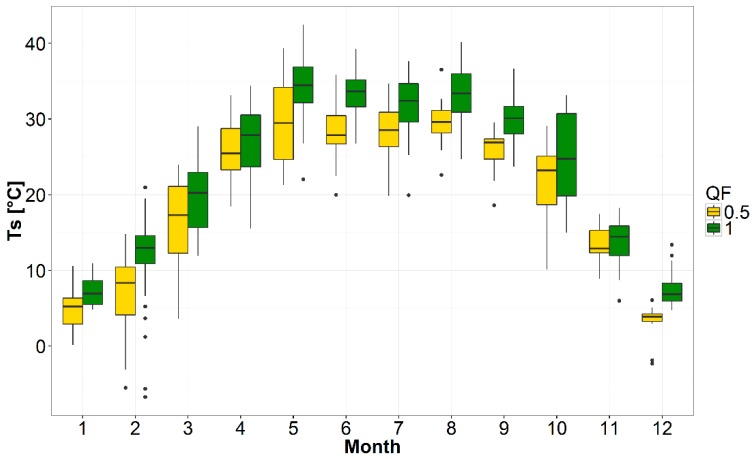
Values of estimated *T_s_* over the two eddy towers for months and QF.

This is further exacerbated by the fact that the ratio between the areas cultivated with winter and summer crops is variable from year to year (Figure A1 in Appendix A). For example, in the case of Livraga 2012, 250 m *NDVI* time series highlight the effect of neighboring winter wheat cultivations on the satellite signal (Figure 3—bottom panel). This is further evident on the 1 km *NDVI* time-series, which in 2012 are found to be quite different from what would be expected for a summer-crop area (Figure A2 in Appendix A).

### 4.3. Spatially Distributed Estimation of EF_d_

**Figure 6:**
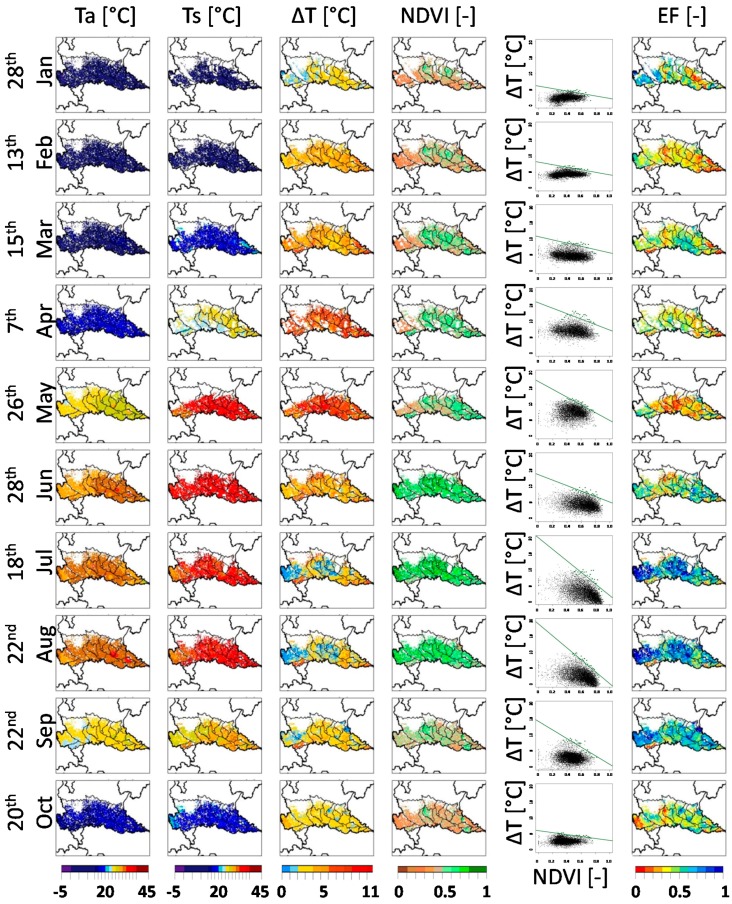
Daily maps of *T_a_* (first column), *T_s_* (second column), *ΔT* (third column), *NDVI* (fourth column) and *EF_d_* (last column) for one representative clear sky date for each month of the year 2010; Overlaid black lines show boundaries of irrigation districts and Lombardy region. White areas are masked out for cloud cover, non-agricultural areas or being outside the Lombardy region. For the same dates are shown, on the second to last column, the daily scatter plots in the *ΔT* vs. *NDVI* feature space.

Figure 6 shows examples of daily *T_a_*, *T_s_*, *ΔT*, *NDVI* and *EF_d_* maps for 10 representative clear-sky dates in 2010. No maps are showed for December and November because no cloud-free data were available. *T_a_* and *T_s_* maps highlight the climatic variability of the study area, with cold winter months, mild spring and hot summer; *ΔT* maps show the highest values between April and May, in relation to the presence of vast bare soil areas associated with increasing incoming radiation and temperature. *NDVI* maps depict the growing season of the major summer crops, with the peak of vegetation greenness between June and August (*NDVI* > 0.5). They also highlight areas where forage and winter wheat are cultivated (October to February), mainly in the central/eastern regions of the study area. The fifth column of Figure 6 shows the *ΔT* vs. *NDVI* scatterplots derived from the input maps and used for *EF_d_* computation, with the dry-edge shown as a green line. It can be observed that the dry-edge slope and position show a strong seasonal variability due to the succession of warm months with pixels characterized by *ΔT* above 10 °C and cold months when the difference between soil and air temperature is usually low (see Appendix C for a more detailed analyses of dry-edge seasonal variability).

*EF_d_* maps are shown in the last column, with lower *EF_d_* areas (drier crop conditions) in orange/red colors, and high *EF_d_* area (well-watered areas) shown in blue colors. Higher *EF_d_* values are obtained in summer months, when evapotranspiration is maximum and the well-watered full vegetated pixels are close to the wet edge in the *ΔT* vs. *NDVI* scatter plots. Districts where rice is the main crop (see CUAA map in Figure 1) are constantly characterized by high *EF_d_* values in summer, because paddies are flooded and never face water stress. Similar summer patterns are obtained in most of the corn districts, because corn is usually well irrigated by the widespread irrigation system of the region.

White areas inside the study area in Figure 6 are due to masked pixels (arable land mask or cloudy conditions), which is more frequent in January to April. Low quality pixels can also be present, to a less extent, during the summer season (Appendix B) thus reducing the available number of reliable *EF_d_* estimations. The use of an aggregated weekly moisture indicator (*EF_w_*), reduces the likelihood of missing information and is therefore more suited for analyzing the overall seasonal trend of crops conditions. This also allows the comparison between different years to highlight inter-annual variations due to unfavorable years characterized by water scarcity or biotic/abiotic stress.

### 4.4. Spatiotemporal Patterns of EF Weekly Indicator

**Figure 7:**
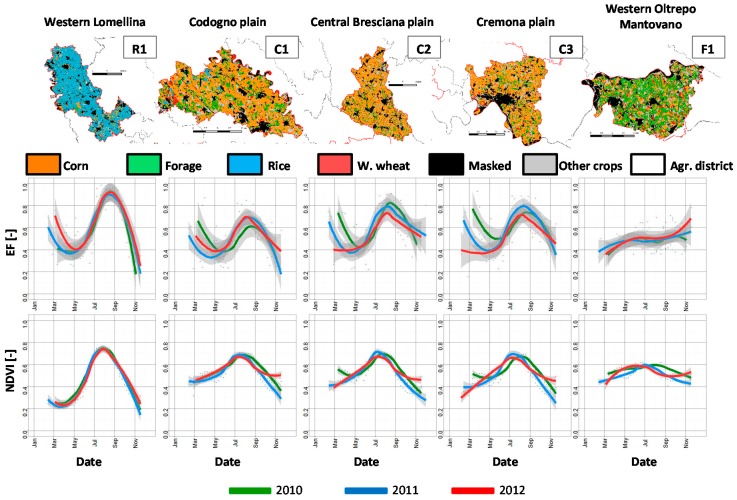
Average weekly *EF* and *NDVI* for three years (2010–2012) over five agronomical districts characterized by different main agricultural land uses. Colored lines correspond to smoothing splines of weekly averages with 95% confidence intervals overlaid in grey The top row shows the major crop types of the districts as extracted from the CUUA land use map.

Figure 7 shows *EF_w_* and average weekly *NDVI* over five agronomical districts in the study area (bold red polygons in Figure 1) from 2010 to 2012.

*NDVI* and *EF* profiles are smoothed with a Generalized Additive Model (GAM) in order to better highlight seasonal trends. Both *NDVI* and *EF_w_* show clear seasonality in the districts dominated by rice (R1) and corn (C1, C2 and C3), with highest values between July and August. Only district F1 (Western Oltrepo Mantovano) shows no seasonal behavior due to the dominance of meadows and Alfalfa (*Medicago sativa* L.) fields (green areas in the CUUA map). These perennial cultivations have a long growing cycle and they are harvested for fodder more than once in a year [83]. In this area, the variability in forage management, the lack of a widespread irrigation system and the presence of winter wheat (red spots in the CUUA map) are the reasons for lower summer *NDVI* and *EF* compared to the other districts.

The rice district (R1) shows the highest summer values of *NDVI* and *EF_w_*, and the lowest inter-annual variability; indeed, rice does not face water stress because it is traditionally grown in flooded conditions [84]. It also shows lower values at the end of the season compared to corn districts, where after corn harvesting stubbles are left over the fields, and plants tend to re-germinate thus increasing *NDVI* and *EF*. Moreover, rice is typically cultivated as single crop [85] thus leading to lower values of *NDVI* and *EF* at the beginning of the season.

*EF_w_* time series over the corn districts (C1, C2 and C3) show high values during the summer season since corn is usually well irrigated and hardly faces water stress. *EF_w_* is however lower and more variable from year to year than in the rice district. In particular, for C2 (Central Bresciana plain) and C3 (Cremona plain) districts *EF_w_* values after July are lower in 2012 compared to other years (Figure 7). These two districts were characterized in 2012 by low and erratic rainfall that affected crop production. Similar results were achieved by Abbas et al. [86] in China by analyzing a multi-temporal spatially averaged drought indicator obtained from MODIS *T_s_* and *NDVI*.

**Figure 8:**
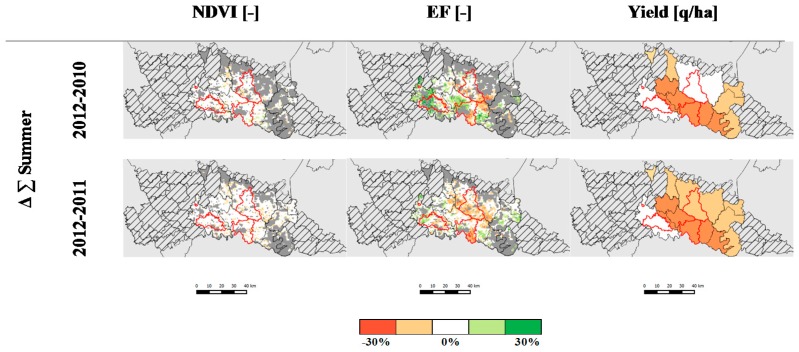
Percentage of the inter-annual difference (*Δ∑_Summer_* [%]) of cumulated *NDVI*, *EF* and Yield. Dark grey color shows areas with no data (cloud cover), grey lines mask agronomic districts where corn covers less than 60% of agricultural surface. Red polygons are the corn districts analyzed (C1, C2 and C3).

Figure 8 shows inter-annual difference, 2012 vs. 2010 and 2012 vs. 2011, of weekly *NDVI* and *EF* values cumulated from June to August (*Δ∑_Summer_*) and maize yield for the corn agronomical districts. Cumulated *EF* shows areas with a major decrease from 2011 and 2010 to 2012, up to −30%. While the corn yield difference maps confirm this difference, no clear difference is appreciable in the *NDVI* Δ∑_Summer_ maps (Figure 8). This result suggests that summer cumulated *NDVI* is less sensitive to water stress periods compared to *EF*; indeed, only prolonged water stress conditions significantly affect crop biomass (i.e., *NDVI*). On the contrary, the *EF* indicator is more sensitive to transient unfavorable conditions since surface temperature promptly increases with respect to air temperature (due to rapid adjustments in stomata closure) if crop water content decreases due to decreased water availability [87].

Results of ANOVA and post-hoc Tukey test are showed in Table 3a. Average (μ) cumulated *EF* in 2012 over C2 and C3 corn districts is statistically lower compared to the other years (α = 0.001), suggesting that corn production has faced a remarkable yield loss due to local dry conditions and scarce water availability for irrigation. No decrease of cumulated *EF* is observed over the Codogno plain district (C1) in 2012. The post-hoc test does not highlight statistically significant differences of the cumulated *NDVI* in any of the districts. This suggests that *EF* might be a more sensitive indicator for identifying changes in crop production related to water shortages. Compared to 2001–2011, average 2012 corn yield shows a stable value over C1 and a significant change over C2 (−5 q/ha) and C3 (−21 q/ha) districts (Table 3b), in agreement with *EF* indicator.

## 5. Conclusions

In this work, the triangle method was applied to retrieve daily *EF* estimates and a weekly indicator of surface moisture status (*EF_w_*) in Northern Italy. Input data include (i) daily *T_a_* maps derived from the interpolation of meteorological stations data, (ii) daily *T_s_* maps derived from MODIS Land-Surface Temperature product, and (iii) *NDVI* maps derived from temporal interpolation of 16-day MODIS Aqua and Terra vegetation indexes products. The main advantage of this method lies in its reliance on just few input spatial data, which makes it suitable for large areas and operative implementation.

Reliability of the estimations depends on AOI characteristics ([23,43,44], see Section 1.1 for details) and accuracy of the input temperature data [21]. The analyzed area, thanks to its great variety of crops with different water managements allows the computation of reliable triangular shape scatterplots in summer, when drought events could occur.

Availability of *T_a_* data simultaneous to satellite overpass is fundamental to compute the wet edge. In our study, interpolated *T_a_* maps showed a more than satisfactory agreement with field measurements, thanks to the dense meteorological stations network available and by the flat topography of the area. These favorable conditions are not frequently met worldwide, but alternative sources for *T_a_* estimation could be exploited, such as using data provided by the European Centre for Medium-range Weather Forecasts (ECMWF) [2] or exploiting an empirical method based on *T_s_*, NDVI and albedo datasets as tested by Sun et al. [76].

The accuracy of MODIS-derived surface temperature (*T_s_*) is greatest for high quality satellite observations (QF1) while intermediate-quality data provide underestimated *T_s_*. Discarding pixels with lower quality is therefore necessary to guarantee reliable *EF* estimates even if the removed data introduce spatial gaps. In many cases, data might not be enough to represent the variability of surface conditions required for estimating the dry edge’s parameters, making *EF* estimation unreliable or impossible on some days. The use of a weekly indicator (*EF_w_*) copes with these limitations, as averaging daily values reduces the impact of both noise in instantaneous estimates and gaps due to unfavorable atmospheric conditions. Moreover, the QA dataset showed that, in the dry spring/summer months, a sufficient number of good-quality observations was available for the computation of a reliable weekly-averaged *EF*. In particular, within the most crucial period for drought crises (from June to August), at least one clear sky satellite image every 3 days was on average available, with a peak of 90% of cloud free observations in July, therefore assuring operational conditions for crop monitoring.

Comparison of the *EF_w_* maps against *in-situ* data showed that estimates are generally in line with weekly-averaged EC measurements, with performance comparable with those of previously published works relying on coarse spatial resolution satellite data. Yet, the simplification of the triangle model, such as the assumption of homogeneous conditions of surface available energy at the time of satellite overpass, leads to a bias in *EF* estimates especially at the regional scale [21].

The agreement between MODIS-derived and field-measured *EF* is also dependent on the difference between MODIS spatial resolution and EC footprint: indeed, this difference could be enhanced in fragmented and heterogeneous agricultural landscapes, where several crop types combined with different agronomic practices are interspersed in a small territory. Moreover, the intra-pixel landscape heterogeneity can lead to *EF* uncertainties since the methodology involves an assumption of uniform aerodynamic properties and physiological behavior of the surface [23].

While more accurate estimates could be achieved by using higher spatial resolution thermal imagery (e.g., de Tomás et al. [23]), satellite sources for this kind of data are currently characterized by longer revisiting time (e.g., 16 days for Landsat 8), that is not sufficient for repeated monitoring along the crop season. Unfortunately, new satellite sensors such as the Sea and Land Surface Temperature Radiometer (SLSTR), onboard ESA Sentinel 3, has thermal infrared bands with the same spatial resolution as MODIS. This limitation however doesn’t prevent the operational use of the *EF_w_* indicator, whose scope is to support management at regional/district scale with a spatially distributed proxy of surface moisture conditions, rather than to assess water needs at field level.

Indeed, at the agricultural district scale, *EF_w_* showed temporal variations consistent with the agro-practices for the major crops in the study area (rice, forages and corn), as well as being correlated to crop production variability induced by water scarcity (i.e., the reduction of corn yield in 2012). These analyses suggest that *EF_w_* can provide information on crop conditions complementary to *NDVI*, which is commonly used as a proxy of annual biomass production [88]. *EF_w_* could be a useful indicator for improving agro-meteorological bulletins providing information on crop status during the growing season at regional scale, allowing to early highlight unfavorable conditions for crop yield, and potentially to implement mitigation activities.

Future work will be dedicated to estimating and analyzing the proposed indicator over the entire MODIS archive (2001–2016) in order to assess its robustness for identification of other drought events occurred in the past decade, among which the ones of 2003 and 2015.

## Figures and Tables

**Table 1:** Indices of agreement between estimated and measured *T_a_* and *T_s_*; for surface temperature the accuracy metrics are provided for high (QF1) and low (QF0.5) quality levels.

Variable	QF	N	Intercept	Slope	*p* value	r^2^	ME	RMSE	rRMSE
T_a_ [°C]	/	713	−1.36	1.00	<0.001	0.95	1.37	2.21	35.14
T_s_ [°C]	0.5	243	0.84	1.06	<0.001	0.74	−2.38	4.98	22.68
T_s_ [°C]	1	649	0.95	0.98	<0.001	0.74	−0.48	3.72	14.32

**Table 2:** Indices of agreement between weekly in situ and satellite *EF_w_*. In bold the best values.

Station	Year	N	Intercept	Slope	r^2^	ME	RMSE
Landriano	2010	10	0.37	0.18	0.07	−0.18	0.22
Landriano	2011	14	0.22	0.56	0.41	−**0.05**	**0.16**
Livraga	2010	9	−0.32	0.79	**0.52**	−0.47	0.48
Livraga	2011	15	0.05	0.56	0.49	−0.21	0.28
Livraga	2012	10	0.23	0.44	0.18	−0.18	0.23
All	All	62	0.16	0.46	0.21	−0.2	0.28

**Table 3:** (**a**) Results of ANOVA and post-hoc Tukey test (α = 0.001) for summer cumulated *EF* and *NDVI* among years (2010–2011) (n = cardinality; μ: district average value); (**b**) official annual corn yield statistics for the 2010–2012 period and last ten years average (2001–2011).

			Codogno Plain			Central Bresciana Plain			Cremona Plain		
			C1			C2			C3		
			2010	2011	2012	2010	2011	2012	2010	2011	2012
(**a**)	$\sum_{Jun}^{Aug}EF_{w}$ [-]	Post-Hoc group	b	a	a	a	a	b	b	a	b
		μ	6.59	7.75	7.72	8.72	9	8.14	8.29	8.9	8.12
		n	229	232	228	283	359	355	231	255	248
	$\sum_{Jun}^{Aug}NDVI_{w}$ [-]	Post-Hoc group	a	a	a	a	a	a	a	a	a
		μ	8.04	7.93	7.71	8.16	7.86	7.54	7.74	7.94	7.55
		n	231	240	228	131	263	230	235	264	248
(**b**)	Yield [q/ha]	Average _2001–2011_		123			120			117	
		Yearly	120	120	120	120	130	115	120	120	96

**Figure A5:**
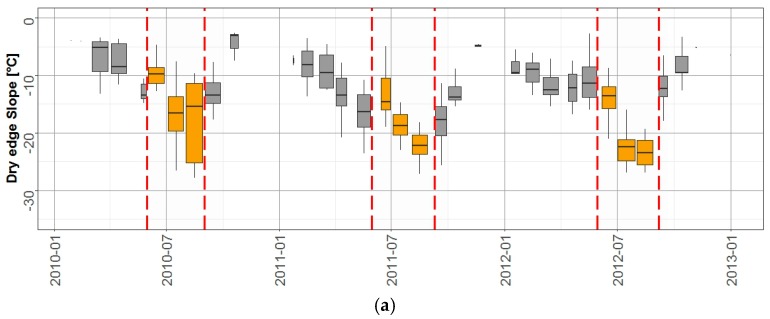

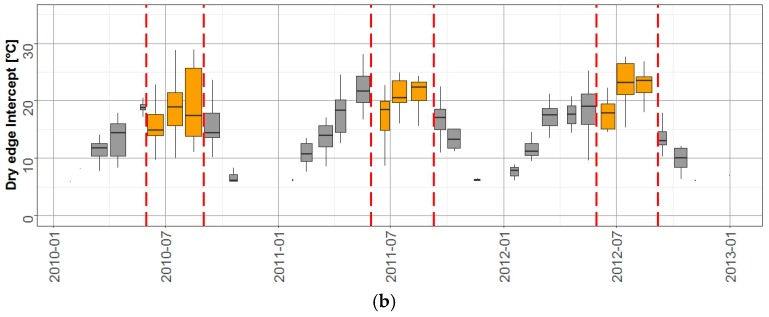
Monthly statistics of the slope (**a**) and intercept (**b**) of the dry edge estimated from the daily scatter plots from 2010 to 2012. Red dotted lines and yellow boxes highlight the summer season (June to August) of each year.

## Appendix B

Table A1 shows indices of agreement between in situ and estimated air and surface daily temperature. Compared to Table 1, regression parameters are divided by site and year. Compared to *T_s_*, *T_a_* data show more homogeneous goodness of fit measures across sites and years.

ME values show that *T_s_* estimates labeled as good quality (QF1) are characterized by a lower underestimation error compared to low quality data (QF0.5) in all sites and years except Landriano 2010 (ME = 1.03 and 2.69 for QF0.5 and QF1 respectively). This station was the only case where eddy data were available in wintertime, when satellite estimation of *T_s_* could be more problematic; yet, water stress monitoring is less meaningful in wintertime.

**Table A1:** Indices of agreement between estimated and measured *T_a_* and *T_s_* divided by sites and years; for surface temperature the accuracy metrics are provided for high (QF1) and low (QF0.5) quality levels.

Data	QF	Site	year	N	Intercept	Slope	*p* Value	r^2^	ME	RMSE	rRMSE
*T_a_* [°C]	/	Landriano	2010	233	−0.77	0.88	<0.001	0.96	2.94	3.61	17.33
			2011	109	−0.36	0.96	<0.001	0.99	1.35	1.42	31.92
		Livraga	2010	108	1.37	0.96	<0.001	0.98	−0.26	0.62	46.99
			2011	146	0.56	0.96	<0.001	0.99	0.53	0.71	47.64
			2012	117	0.36	0.95	<0.001	0.99	0.79	0.96	34.07
*T_s_* [°C]	QF0.5	Landriano	2010	53	0.77	0.92	<0.001	0.85	1.03	3.50	15.68
			2011	60	7.88	0.88	<0.001	0.75	−4.73	5.32	23.60
		Livraga	2010	48	−1.63	1.14	<0.001	0.66	−2.54	4.36	18.75
			2011	46	8.47	0.86	<0.001	0.59	−4.59	5.57	24.53
			2012	36	5.95	0.96	<0.001	0.46	−4.80	7.62	43.46
*T_s_* [°C]	QF1	Landriano	2010	112	0.42	0.88	<0.001	0.92	2.69	3.91	15.59
			2011	138	2.72	0.95	<0.001	0.67	−1.02	2.66	10.04
		Livraga	2010	114	3.37	0.92	<0.001	0.51	−0.87	3.56	14.13
			2011	147	7.99	0.84	<0.001	0.75	−3.05	3.77	14.15
			2012	138	6.51	0.78	<0.001	0.45	0.44	4.44	17.05

Figure A3 shows the proportion of the daily quality flags falling in the three classes (see Section 2.2) for each month from 2010 to 2012 at the two EC towers. In winter months (November to February), more than 70% of daily *T_s_* data are missing (QF0) due to frequent cloud cover, but in the spring-summer period (March to September) at least 60% of the *T_s_* data per month are produced (QF0.5, QF1), with an even greater proportion in the driest months of the year, which are of key relevance for water stress monitoring (peak of 90% of cloud free dates in July). Cardinality of available *T_s_* data is consistent over sites and years.

Boxplot in Figure A4 shows the statistics of *T_s_* satellite estimation per months for good (QF1) and low quality data (QF0.5). Either time series shows a coherent temporal behavior. However, the medians of the boxes show that QF0.5 data always have lower values because of the well-known atmospheric interference. The use of QF0.5 data could lead to a misleading computation of ∆*T*, as is the case.

## Appendix C

Seasonal trends of the parameters of the regression line representing the dry edge are shown in Figure A5. Monthly statistics of the slope and intercept are plotted against time with a box-plot representation. The summer months, from June to August, when temperatures are higher and water shortage could became an issue, are highlighted by yellow boxes in the graphs and delimited by red dotted lines.

With the onset of the growing season in late spring and the beginning of the summer period, dry edge becomes gradually steeper reaching average minimum slope between July and August as also displayed by scatter plots in Figure 6. Dry edge lines with high intercept and low slopes indicates dates characterized by greater difference between air *T_a_* and surface *T_s_* temperature, a thermal condition typical of hot months when stressed pixels are present in the area together with well-watered areas; in these months of the year a wider range of thermal conditions of crops is reached.

Winter and spring months show, on average, dry edges with smaller slopes and intercepts close to zero due to both lower absolute temperature values and smaller difference between surface and air temperature (*ΔT*). During this time of the year in the study area, atmospheric water demand, hence potential evapotranspiration, is very low, in fact winter crops are not irrigated. Clearly, months when crop biomass is low or vegetation absent, the full range of thermal conditions are not covered and the estimated dry edge could be far from the theoretical one [6,23,32,76].

The year 2012 displays the summer months with the highest intercepts, in particular in July (median value for intercept equal to 21 °C) consistent with the particular dry weather conditions of this year. The seasonal behavior displayed in the boxplots fits with the typical average climate condition of the study area [2] and are consistent with results from previous work carried out in different ecoregions [20,36,37,38,76,89,90].
